# Racial and ethnic data justice: The urgency of surveillance data disaggregation

**DOI:** 10.1016/j.dadr.2022.100082

**Published:** 2022-07-27

**Authors:** Camila Gelpí-Acosta, Manuel Cano, Holly Hagan

**Affiliations:** LaGuardia Community College, City University of New York, USA; School of Social Work, Arizona State University, USA; School of Global Public Health, Center for Drug Use and HIV/HCV Research, NYU, USA

## 1. Background

Across industrialized countries, epidemiology has historically relied upon racial and ethnic classifications to characterize the populations it describes (Boykin and Williams, 2010). In the United States (US), this is true across federal, state, and local public health institutions. Hence, most community-based organizations leading the fight against diseases such as HIV, Hepatitis C (HCV) and the overdose epidemic can only mirror these categories at the frontlines (i.e., harm reduction outreach workers, medical personnel, case managers and social workers across syringe services programs, opioid agonist therapy centers and overdose prevention centers). Across communities burdened by HIV/HCV and drug overdose, and particularly socioeconomically disadvantaged people of color in the US, these frontline workers are medullar entry points to our care continuums. Limitations on how these data are organized and presented can impact public health policy and frontline work efficacy.

## 2. The overarching need for racial and ethnic data disaggregation

The disaggregation of racial groups such as Non-Hispanic (NH) White, Asian, and NH Black, shows that NH Black people in the US are disproportionally more likely than NH Whites to be infected with HIV and to suffer drug overdoses. This is crucial information that leads to the development of policies and programs, at the frontline level, to tackle these vulnerabilities more precisely. But this is far from perfect. Not all people lumped into the NH Black category are the same, and this is perhaps partly why prevention strategies that rely on this category to understand those they aim to help haven’t been as effective. Persistently high rates of HIV among NH Blacks showcase this fact. The same holds true for those who fall under the Asian racial category, a group so heterogenous and disparate (~50 Asian countries) that forces us to question the epidemiological usefulness of this category. Recently, New York State mandated the disaggregation of Asian American Pacific Islander data, including health data, to better comprehend their needs and to mitigate the vulnerabilities of this diverse group (Coalition for Asian American Children and Families, 2021). The same mandate should be considered for other racial/ethnic groups: NH Blacks, Latinx and NH Whites.

Just as with Asians, under the NH Black rubric we find people from an array of heritages and cultures that render this classification unhelpful. In 2019, at least 10% of people who identify as NH Black were foreign-born, a percentage that is rapidly growing in the US (Tamir, 2021, PEW Research Center). But even US-born NH Black people with Haitian heritage are culturally different to their Jamaican counterparts. While Black African Americans are also markedly different to these groups, they are all lumped under the NH Black category. Not surprisingly, a wide variance of people of Latinx ancestry are also lumped under the ethnic Hispanic/Latinx category. Arguably, the term Latinx is a riposte to the Eurocentric Hispanic term, which incorrectly assumes all Latinx people speak Spanish and/or are of Spanish descent. But the message this approach sends is: all Asian people are the same; all Black people are the same; and all Latinx people are the same. This is structural racism because it helps perpetuate the disparate burdens of HIV, HCV, and drug overdoses affecting racial minorities in the US.

But the need for ethnic data disaggregation also applies to NH Whites. Whilst only 3.97% of NH Whites in the US are foreign born (US Census Bureau, 2020), ethnic variance (i.e., Irish, British, Italian, Spanish, Russian, etc.) can be associated with different health outcomes. This includes people who use drugs (PWUD), and research has shown that PWUD from the former Soviet Union living in New York City (NYC) have worse health outcomes associated with their drug use than their non-former Soviet Union NH White counterparts, and that these outcomes are directly associated with cultural aspects unique to this ethnic group (Guarino et al., 2012). Currently, the Centers for Disease Control and Prevention report the presence of Xylazine (a sedative used by veterinarians) is threatening the health of PWUD in the Northeast, and particularly of NH Whites (Kariisa et al., 2021). And yet, the latter classification prevents the detection of ethnic vulnerability variance that could potentially help improve prevention efforts at the frontline level. Hence, ethnic data disaggregation is needed across racial classifications.

## 3. People who inject drugs surveillance

In cities such as NYC, Latinx people constitute at least half of the population of people who inject drugs (PWID). Of these, > 95% are Puerto Rican, distantly followed by Dominicans (Alexis Rivera, MPH, NYC Department of Health and Mental Hygiene, National HIV Behavioral Surveillance/NHBS, personal communication, February 2022). Since 2009, the NHBS study with PWID in NYC has disaggregated Latinx data and has paid attention to place of birth. Ergo, we know ~50% of Puerto Rican PWID in NYC were born (and likely started injecting drugs) in Puerto Rico, a context fundamentally different to NYC that requires culturally appropriate lenses to understand their HIV, HCV, and overdose vulnerabilities (Gelpí-Acosta et al., 2021). While similar ethnic distributions exist among PWID in other Eastern Seaboard cities and in Chicago, surveillance is yet to reflect it. If we understand the underlying causes of risk, we can learn how to mitigate them. But knowledge is power *only if* it guides surveillance.

## 4. Overdose surveillance

Generally, across federal, state, and local jurisdictions overdose surveillance data are not disaggregated by ethnicity or place of birth, a baffling fact when considering ~14% of the US population is foreign born (not counting those born in Puerto Rico, a territory of the US) (US Census Bureau, 2019). This remains so even when compared to NH Whites, NH Black and Latinx people are the most vulnerable to drug overdoses. For instance, in analyzes of national data, 2020 represented the first year since 2001 when drug overdose mortality rates in the NH Black population overall surpassed the NH White population (Hedegaard et al., 2017, 2021). Nonetheless, rates disaggregated by nativity and sex at birth indicate that US-born NH Black men have been dying of drug overdoses at higher rates than US-born NH White men since 2017 (Cano and Sparks, 2022). Similarly, within the Latinx umbrella, Puerto Ricans are the most overdose vulnerable across several urban areas in the US (Cano and Gelpí-Acosta, 2020, 2022), a fact recently confirmed in NYC by a New York City Department of Health report, which showed that the overdose death rate was 32.7 among NH Whites, 38.2/100,000 NH Black New Yorkers and 43.6/100,000 among Puerto Rican New Yorkers, as compared to the aggregated rate of 33.6/100 K Latinx (New York City Department of Health and Mental Hygiene, Bureau of Alcohol, Drug Use, Prevention, Care and Treatment, 2020). We need surveillance systems that capture ethnic variance across race groups to help guide frontline harm reduction efforts to prevent drug overdoses more effectively.

## 5. HIV surveillance

**Fig. 1.**
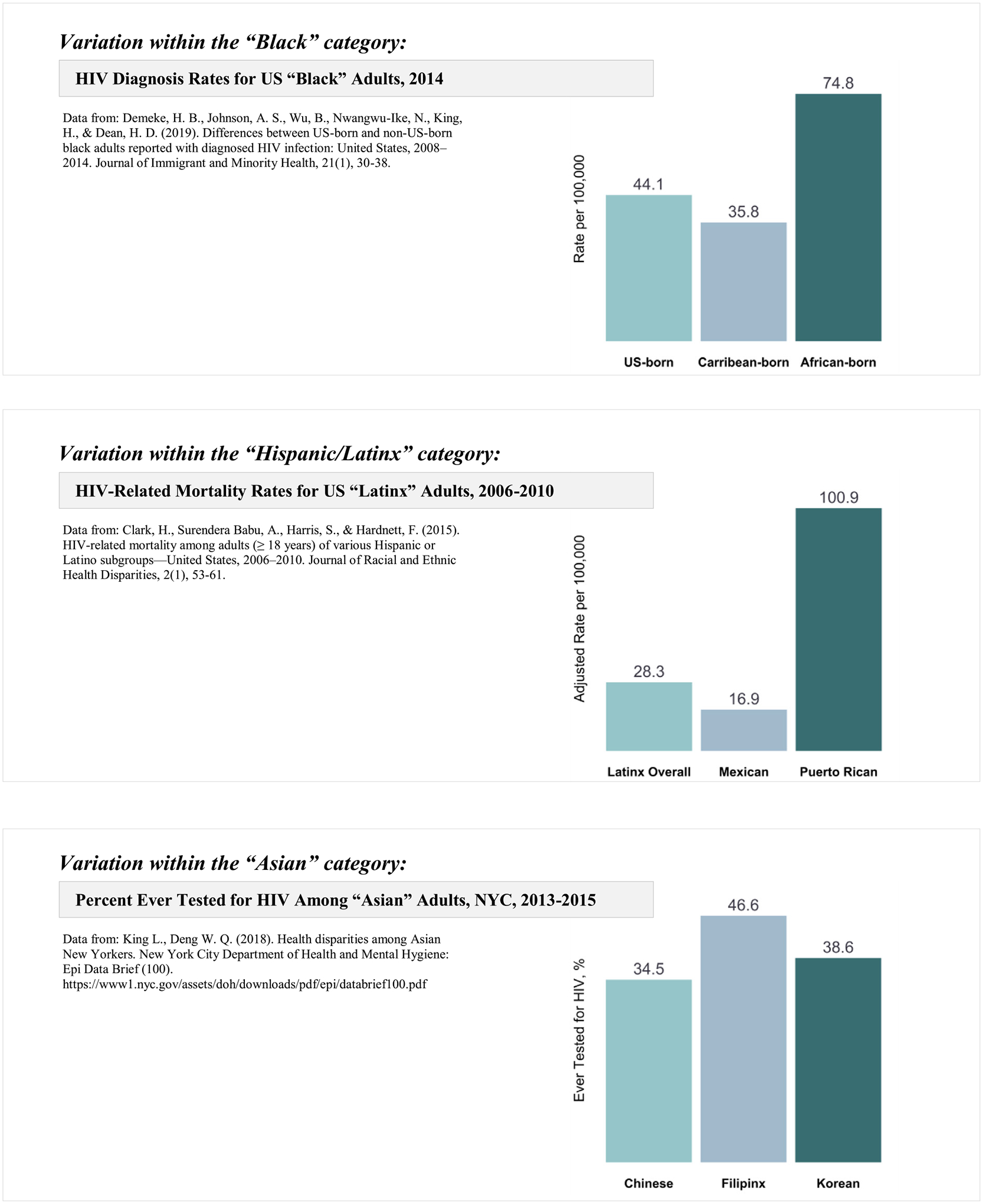
The importance of health data disaggregation beyond broad Racial/Ethnic categories: three examples with HIV data from existing literature.

HIV surveillance data are not disaggregated by ethnicity and place of birth, and there is ample evidence that it should. For instance, while data for the overall Hispanic/Latinx population indicated that just 6.5% of new HIV diagnoses among males were attributed to injection drug use, disaggregation by place of birth revealed that injection drug use accounted for one in four (24.9%) new HIV infections among men born in Puerto Rico (Gray et al., 2015). Thus, a broad category such as Hispanic/Latinx hides the wide variation in the method of HIV transmission, consequently hindering efforts to mitigate the spread of HIV. As Fig. 1 portrays, a broad category such as Black also hides dramatic differences in rates of new HIV diagnoses; disaggregation by place of birth (not by ethnicity) revealed that rates of US HIV diagnosis were higher in African-born Blacks than US-born Blacks (Demeke et al., 2019). Fig. 1 also shows how the Hispanic/Latinx category hides the disproportionate HIV vulnerability of Puerto Rican people when compared to other Latinx groups (Clark et al., 2015), and the Asian category similarly obscures significant variance across Asian groups and the heightened HIV vulnerability among Filipinx (King and Deng, 2018). Moreover, in NYC disaggregating by place of birth indicated that the US-born NH Blacks experienced the highest rate of new HIV diagnoses, at 109.48 per 100,000 residents, followed by immigrants from Haiti (70.46 per 100,000) and the West Indies (43.19 per 100,000), dwarfing the rates of US-born Whites (19.10 per 100,000) (Hoffman et al., 2012). Again, these surveillance limitations render partially blind HIV prevention efforts at the front lines. We cannot end HIV/AIDS if we continue to disregard people’s cultural backgrounds which are important determinants of health, as well as the differences within groups in terms of the specific structural barriers most implicated in poor health outcomes.

## 6. HCV surveillance

HCV surveillance is plagued with the same limitations, lumping people from > 30 Latin American countries under the Hispanic/Latinx category effectively disregarding diversity within these communities (Centers for Disease Control and Prevention, 2019). For example, reliance on this Hispanic/Latinx category hides that Puerto Ricans are the most vulnerable Latinx group to HCV infection (via injection drug use) and mortality (Kim et al., 2019; Arasteh et al., 2020). This data void de facto deprives frontline workers from the knowledge necessary to make their efforts more efficacious. In this ethnic group, researchers have identified cultural beliefs such as HCV not being a legitimate health threat (Gelpí-Acosta et al., 2019), which neutralize their risk concerns and render negative health outcomes. An ethnic and place of birth-aware surveillance would enable culturally appropriate prevention and treatment programming, making the HCV elimination goal by 2030 more attainable.

## 7. Conclusions

In this commentary, we highlight the urgency of conducting health surveillance using disaggregated ethnic categories across all racial groups, and to factor place of birth to mirror more closely the composition of our communities. For example: Hispanic/Latinx can be presented instead as Dominican, Mexican, Puerto Rican, etc.; NH Black as African American, Jamaican, Haitian, etc.; and NH White as British, Canadian, Irish, etc. Disaggregation provides a clearer picture of vulnerable communities and helps public health workers target their needs more precisely. Data disaggregation is particularly urgent for PWUD and PWID who are racial minorities bearing the brunt of HIV, HCV, and drug overdoses. We cannot end HIV, HCV, and drug overdoses if we continue to disregard the very people who are at stake in our epidemiology. Disaggregate surveillance data. A more effective public health agenda requires it.
